# Vision-Based Localization System Suited to Resident Underwater Vehicles

**DOI:** 10.3390/s20020529

**Published:** 2020-01-18

**Authors:** Petar Trslić, Anthony Weir, James Riordan, Edin Omerdic, Daniel Toal, Gerard Dooly

**Affiliations:** 1 Centre for Robotics & Intelligent Systems, University of Limerick, V94 T9PX Limerick, Ireland; anthony.weir@ul.ie (A.W.); edin.omerdic@ul.ie (E.O.); daniel.toal@ul.ie (D.T.); gerard.dooly@ul.ie (G.D.); 2 School of Computing, Engineering, and Physical Sciences, University of the West of Scotland, Glasgow G72 0AG, UK; james.riordan@uws.ac.uk

**Keywords:** resident UUVs, localization, subsea navigation

## Abstract

In recent years, we have seen significant interest in the use of permanently deployed resident robotic vehicles for commercial inspection, maintenance and repair (IMR) activities. This paper presents a concept and demonstration, through offshore trials, of a low-cost, low-maintenance, navigational marker that can eliminate drift in vehicle INS solution when the vehicle is close to the IMR target. The subsea localisation marker system is fixed on location on the resident field asset and is used in on-vehicle machine vision algorithms for pose estimation and facilitation of a high-resolution world coordinate frame registration with a high refresh rate. This paper presents evaluation of the system during trials in the North Atlantic Ocean during January 2019. System performances and propagation of position error is inspected and estimated, and the effect of intermittent visual based position update to Kalman filter and onboard INS solution is discussed. The paper presents experimental results of the commercial state-of-the-art inertial navigation system operating in the pure inertial mode for comparison.

## 1. Introduction

In recent years, we have seen the conceptual introduction of resident robotic platforms to underwater inspection, maintenance and repair (IMR) activities within the oil and gas sector. This is being driven by cost saving activities to reduce the levelized cost of energy (LCOE) for the sector and in an effort to compete with emerging sectors such as hydraulic fracturing. Traditionally, subsea IMR activities are carried out using a work-class hydraulic remotely operated vehicle (ROV). Vehicles are tethered and controlled directly from a surface support vessel and generally incur significant expenditures associated with the cost of the vessel and crew. The concept of permanently deployed ROV systems has been in the literature for many years [1]; however, only in recent years have we seen the introduction of these system to commercial marine activities offshore. IKM Subsea [2] were one of the first commercial entities to bring this from the proof-of-concept prototype stage to full operational system enactments. IKM developed their fully electric Merlin UCV ROV, modifying their manipulator protocols along with umbilical connectors for shore-based distance piloting and long-term deployment [3]. This was developed and deployed under a breakthrough ten-year contract for Statoil’s Visund and Snorre B field assets where one resident ROV system was included for deployment onsite [4]. The outputs from this and similar commercial projects [5,6] signalled a justification for resident robots on underwater infrastructure projects, driving a change in how offshore operators manage IMR activities. Resident systems allowed for less personnel offshore and for shared onshore resources, thereby reducing costs and enabling more efficient operations. Additionally, one of the primary benefits to this approach was shown to be the extended operational weather windows which can be achieved through having system deployments available on the seabed at all times.

This extended weather capabilities has hugely significant benefits, not just to oil and gas but also to the offshore wind sector, which has seen the rollout of many large infrastructure projects off European waters in recent years. However, typically within offshore wind and oil and gas, multiple assets can be spread across hundreds of square kilometres of seabed, and a resident vehicle capable of performing an inspection across these multiple distributed assets would be required. This mobility is being addressed through developments in resident autonomous underwater vehicles (AUVs) [7,8]. However, there are many concerns for resident AUV systems suited to offshore wind including major technical building blocks such as autonomy, power, communications, navigation accuracy and connectivity [9].

### Navigation Accuracy over Resident Fields

Traditionally, underwater navigation systems are based on acoustic systems, providing an absolute world coordinate frame position solution with technologies such as Long baseline (LBL) or Ultra-short baseline (USBL) systems [10]. However, acoustic systems suffer from a number of disadvantages such as loss of accuracy in noise-polluted environments, calibration of the units, high system complexity/maintenance, and high error rates. This is far from an ideal solution for resident field systems where noise, maintenance and accuracy are key issues that need to be addressed in achieving cost effective IMR services.

Various research has been completed in the field of navigation sensor fusion [11,12], with modern commercial navigation systems exploiting a number of fused sensor types. A common practice in AUV navigation is to use onboard inertial navigation systems (INS), coupled with a Doppler velocity log (DVL), and additional sensors such as depth sensor and sound velocity probe, ultimately providing a dead-reckoned relative based position solution. However, the concerning issue in this solution is drift over time which in a commercial state-of-the-art system is 0.1% of distance travelled [13,14]. This is not an acceptable error rate for subsea operations requiring close-quarter IMR activities. A solution that is widely used within bathymetric survey is the dual use of LBL acoustic positioning with INS; this eliminates drift and provides a greater level of accuracy in the solution. However, an acoustic system for elimination of drift does not address other issues in underwater resident robotics including noise-polluted environments and high system complexity/maintenance. Vision based navigation have also been recorded in the literature for use in underwater cable tracking [15,16], localization in structured environment [17], and various automation of intervention tasks [18,19]. The field of underwater simultaneous localisation and mapping (SLAM) is very active [20] and has allowed for vision and sonar based navigation in unstructured environments [21]. However, the main concern is that most vision-based techniques require low water turbidity and a relatively close proximity to the target [22,23].

The fact that resident vehicles will operate in partially structured environments consisting of known subsea structures (docking stations, subsea interconnection stations, oil wells, etc.) can be utilised and can offer a means of facilitating a low cost, low maintenance, navigational marker that can eliminate drift in INS solution when the vehicle is close to the IMR target. This research paper discusses the use of an LED based subsea location marker at a known structure position for vehicle position update. The localisation aid provides pose estimation based from active light marker beacons for intermittent resident ROV position update and intermittent drift elimination close to target, where accuracy is required. This paper presents evaluation of the system during trials in the North Atlantic Ocean during January 2019. System performances and propagation of position error are inspected and estimated, and the effect of intermittent visual based position update is discussed.

## 2. Hardware and Experimental Setup

**Figure 1:**
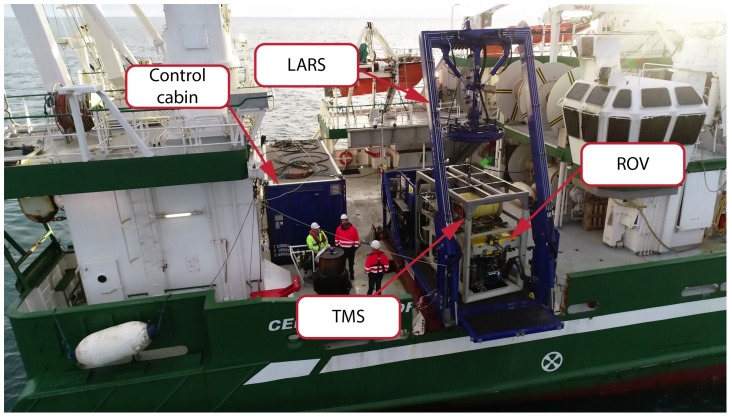
Experimental setup used during the offshore trials in North Atlantic Ocean during January 2019. The system consists of control cabin, A-frame launch and recovery system, ROV and the corresponding tether management system.

The experimental setup, for the trials performed during January 2019 in North Atlantic Ocean, is shown in Figure 1. The system consists of a control cabin, launch and recovery system (LARS), tether management system (TMS), and the remotely operated vehicle. The ROV used for the trials is work class Comanche ROV by Subsea, which is one of the standard ROVs used throughout the offshore sector. The ROV was deployed from the vessel using the launch and recovery system with a corresponding cage type TMS. For initial experiments, the tether management system was deployed to the seabed, acting as a ‘fixed subsea structure’. The light beacons were fixed to the TMS creating an active, light beacon marker that was utilised to provide high-refresh-rate, long-range ROV visual pose estimation.

### 2.1. Light Marker and Camera

**Figure 2:**
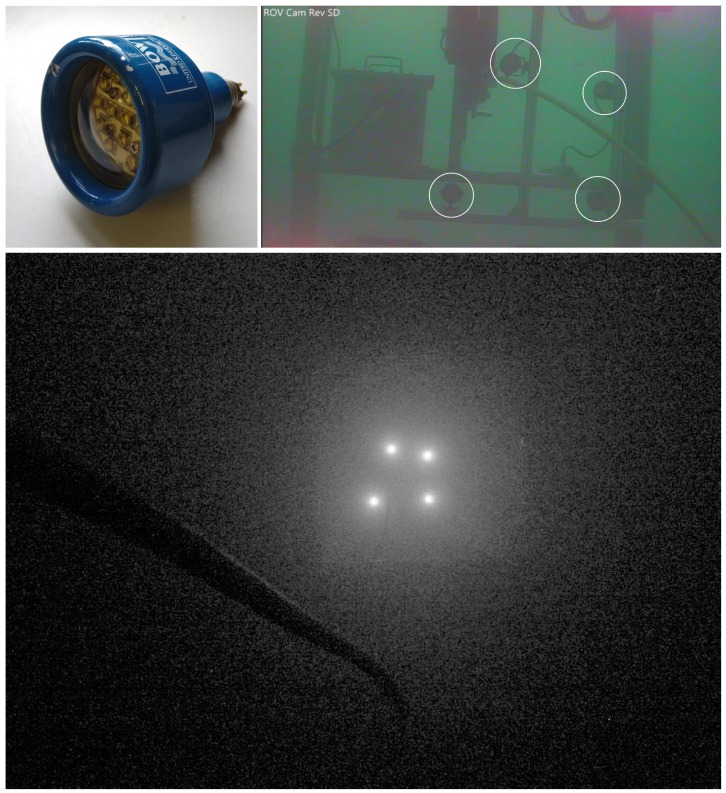
Underwater light used for a light marker attached to the TMS (**top left**); Lights mounted on TMS forming a light marker (**top right**)—a photo taken from a rear ROV camera; the light marker observed from approximately 7 m distance (**bottom**); the photo taken using an image acquisition camera.

The system for visual pose estimation consists of a machine vision camera IDS uEye with Sony 1/1.8” CMOS (IMX265), and with fixed focus Lensagon BM4518S lens. The camera is enclosed in subsea housing with flat port. Four conventional subsea LED lights Bowtech LED-K-3200-DC were used as a light marker. The lights, standard throughout the sector, are shown in Figure 2. Four lights were attached to the tether management system creating a unique light marker with four beacons. To minimise the localization error, the camera used during the experiment must be precalibrated. To achieve highest precision, the best practice is to calibrate a camera on site and, after the camera is calibrated, it is ready to be used for image acquisition. The acquired image is sent to a topside PC where image processing is done. Detailed camera calibration and image acquisition process for the ROV localization, as well as hardware setup, has been presented in [24,25].

### 2.2. The Navigation System

The heart of the ROV navigation system is the state-of-the-art inertial navigational system PHINS 6000. The system is coupled with a DVL Nortek 500, a depth sensor, an ultra-short baseline system Teledyne Ranger 2, and a differential GPS unit Okeanus GPSR-3015G. The technical specifications of the navigational system components are given in Table 1. Navigation filter within the commercial INS unit is Extended Kalman Filter (EKF). The EKF is used to combine and fuse an internal and external sensor data. The GPS unit and the USBL are used for the absolute navigation measurements and position drift corrections. Additionally, the GPS unit is used for initial alignment of the fibre optics gyroscope (FOG) within the INS unit, and for navigation when the vehicle is on the surface, while USBL provides absolute position underwater. Without USBL the ROV position underwater drifts over time.

Section 3 further describes inertial system PHINS 6000, the components of the system, operating modes and propagation of the position error in pure inertial mode.

## 3. Inertial Navigation System PHINS 6000

Commercial inertial navigation system PHINS 6000 was used during the trials. The system presents a gold standard within marine robotics navigation and consists of three major components:

- Inertial measurement unit (IMU) which consists of three fibre optic gyroscopes and three accelerometers,
- Inertial navigation system (INS) resolving inertial measurements and updating position, velocity, and attitude,
- Extended Kalman filter for optimal integration of external and internal sensor measurements.

**Figure 3:**
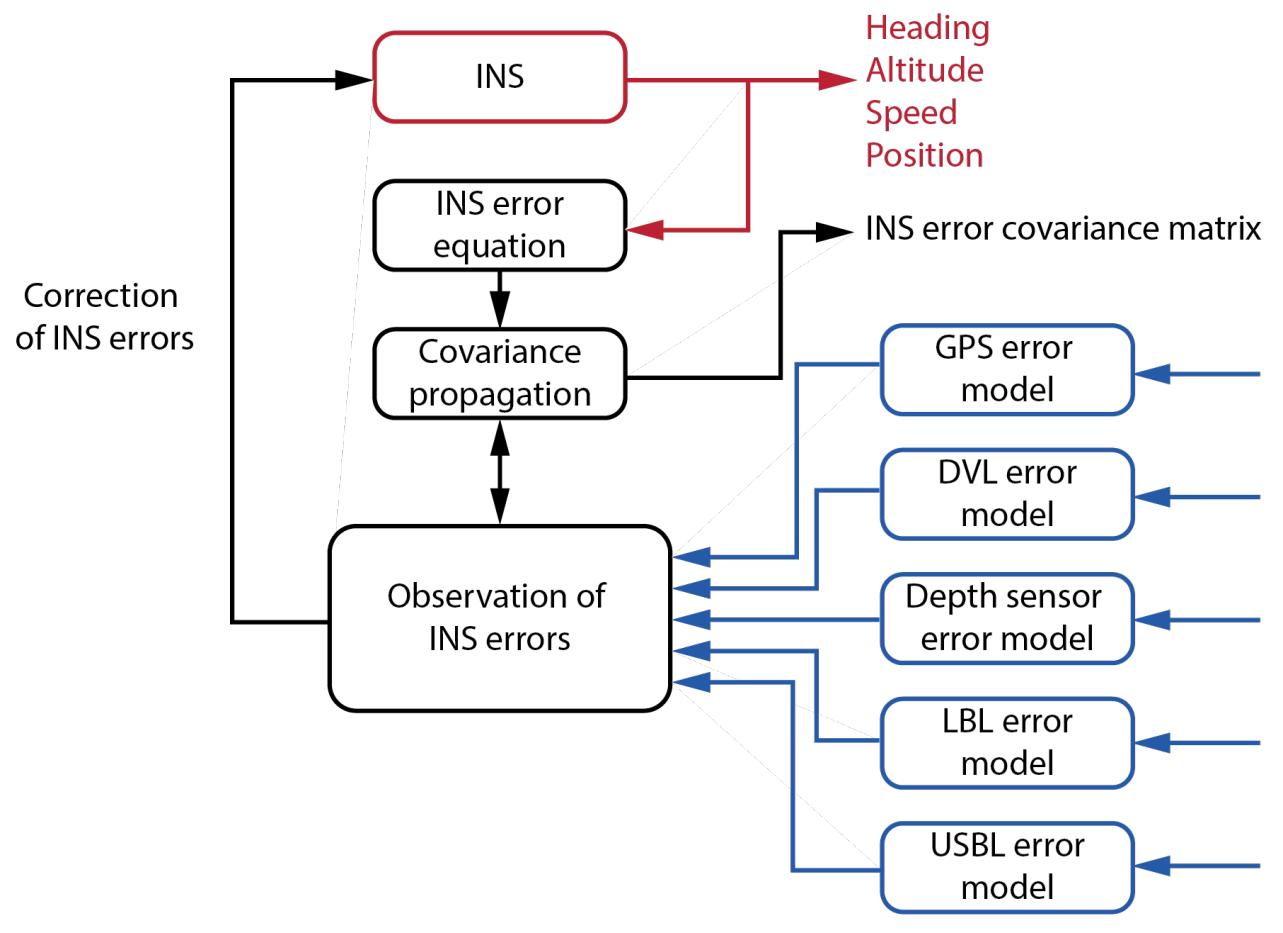
Function block diagram of the Kalman filter.

Figure 3 shows a functional block diagram of the PHINS Kalman filter. If no external sensor data are fed in, the output of the INS unit is based purely on the IMU measurements. These data are used to update coefficients of the error equations. The error equations are used to update the covariance matrix. The INS estimates are compared with the observed external sensor measurements and used as feedback to the INS. In case no external sensor measurement was received, the Kalman filter provides error bound estimates. A software switch separates the external sensors and the EKF. Thus, it is possible to operate the system in different operation modes with a various combination of external sensor data.

### 3.1. PHINS Operating Modes

The navigation system PHINS 6000 operates with various external sensors such as GPS, USBL/LBL, depth sensor, and DVL. The system operates in various modes depending on the combination of external sensors used. The GPS is usually used for initial heading alignment process while on the ship’s deck, and to acquire an absolute, geo-referenced position while ROV is on the surface.

While operating underwater, there are three PHINS operating modes based on the external sensors used:

- INS + USBL/LBL + (DVL),
- INS + DVL,
- INS (Pure inertial).

The best pose estimation performance is achieved while PHINS operates in USBL + INS mode. The USBL provides an absolute position while INS reduces the noise on the USBL position measurements. As shown in Table 2, PHINS position accuracy in this mode is three times better than the accuracy of the standalone USBL system. Throughout the trials, the measured standard deviation of the ROV position, in this mode, was between 0.25 m and 0.3 m at all times. Additionally, DVL can be used in conjunction with USBL and INS measurements to provide additional precision, especially in the case of intermittent USBL signal loss.

Without USBL/LBL absolute position fix, the position measurement drifts over time. The DVL + INS operating mode minimises the drift due to a constant speed over ground measurement. In this mode, the position drift of PHINS 6000 is approximately 0.1% of the travelled distance. Pure inertial mode without external sensor aiding is considered a worst-case scenario. The position error is estimated based on the integration of the IMU data, thus it depends on the quality of the FOG and accelerometers.

### 3.2. Propagation of Errors in Pure Inertial Mode

Position estimation accuracy in the pure inertial mode depends on two factors: (1) the accuracy of the IMU acquired measurements, therefore the accuracy of the accelerometers and FOGs, and (2) an initial position, velocity, and attitude errors obtained after the alignment/position fix. Considering the IMU data are integrated over time, the position error contained within the INS covariance error matrix propagates with time as well. Therefore, assuming the same initial position error in the pure inertial mode, the propagation of the position error is only a function of time. In practice, the initial position error varies mostly due to the quality of the position fix. During few days of the ROV operations, the position error based on USBL position fix was between 0.23 m and 0.57 m. Some of the factors that may influence the quality of the position fix are the strength of the USBL signal, a partial USBL responder occlusion, subsea noise pollution, and distance between transponder and receiver.

**Figure 4:**
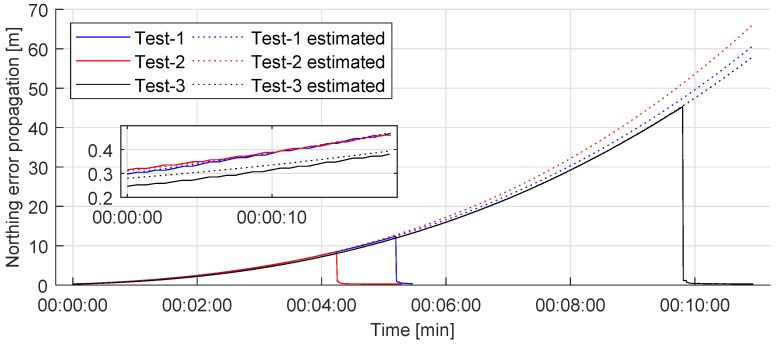
The INS position error propagation in pure inertial mode.

The propagation of position error in the pure inertial mode was further studied. Figure 4 shows the propagation of the measured position error during three tests of various durations. The experiment was performed in the North Atlantic Ocean off the coast of Ireland. Each test started with PHINS operated in the INS + USBL mode. As shown in the magnified section, the initial position error was between 0.25 m and 0.33 m, depending on the test. After the initial position fix was acquired, the INS was set to operate in the pure inertial mode.

The continuous line shows recorded position error propagation over time. During the tests, the ROV was manually piloted while performing a general visual inspection of the seabed. At the end of the test, PHINS was switched back to operate in INS + USBL mode. This reduced the position error to the initial values. Although the ROV paths during the tests were different, the propagation of the error behaves nearly equal. As shown in the figure, the position error is approximately 2 m after 2 min, 12 m after 5 min, and 40 m after 9 min. Since PHINS is a commercial system, the error propagation model is not known, thus it was necessary to measure error propagation and model the error propagation function. The dashed lines in Figure 4 present corresponding 3rd order error propagation functions calculated using least-squares polynomial curve fitting. In case a vision based position fix is acquired at any time throughout the experiment, this function is to be used for the prediction of the position and position error estimates, assuming PHINS continues to operate in the pure inertial mode.

In the next section, the performance of the vision-based pose estimation system used during the trials is to be compared with the USBL data. If comparable, the visually estimated pose data could be used as PHINS position update. Additionally, the estimated position error is to be reduced using intermittent position fix.

#### Additional Considerations

Position error estimates provided by PHINS are based on the INS accelerometers and gyroscopes theoretical models, thus are not accurate physical measurements. Although the relative difference of the initial position error was small across three tests initially, the difference grows exponentially after longer periods of time, as shown in Figure 4. For that reason, the derived error propagation function is considered to describe the behaviour of the system’s error propagation correctly for up to 6 min.

## 4. Visual Pose Estimation

A visual position estimation method for subsea localization is described in this section. Since the method presents an upgrade of the method used during field trials, more detailed explanation of the image acquisition, camera calibration, and pose estimation procedure can be found in [25]. Prior to the pose estimation, the camera was calibrated on site using calibration panel with a 7 × 10 chessboard pattern. The calibration algorithm assumes a pinhole camera model [26]. Calculated lens distortion and camera intrinsic parameters are then used to correct image distortion. The method estimates the relative position between the camera and the marker using a single camera and a marker of known size shown in Figure 2. The proposed solution that consists of four conventional light beacons mounted on the tether management system assembling the active light navigation marker. For the experiment, the TMS is deployed to the seabed and presents a permanently fixed subsea structure. It is assumed that the absolute position of the subsea structure is known in the world frame, therefore the absolute position of the light marker is measured and known.

### 4.1. Image Processing

**Figure 5:**
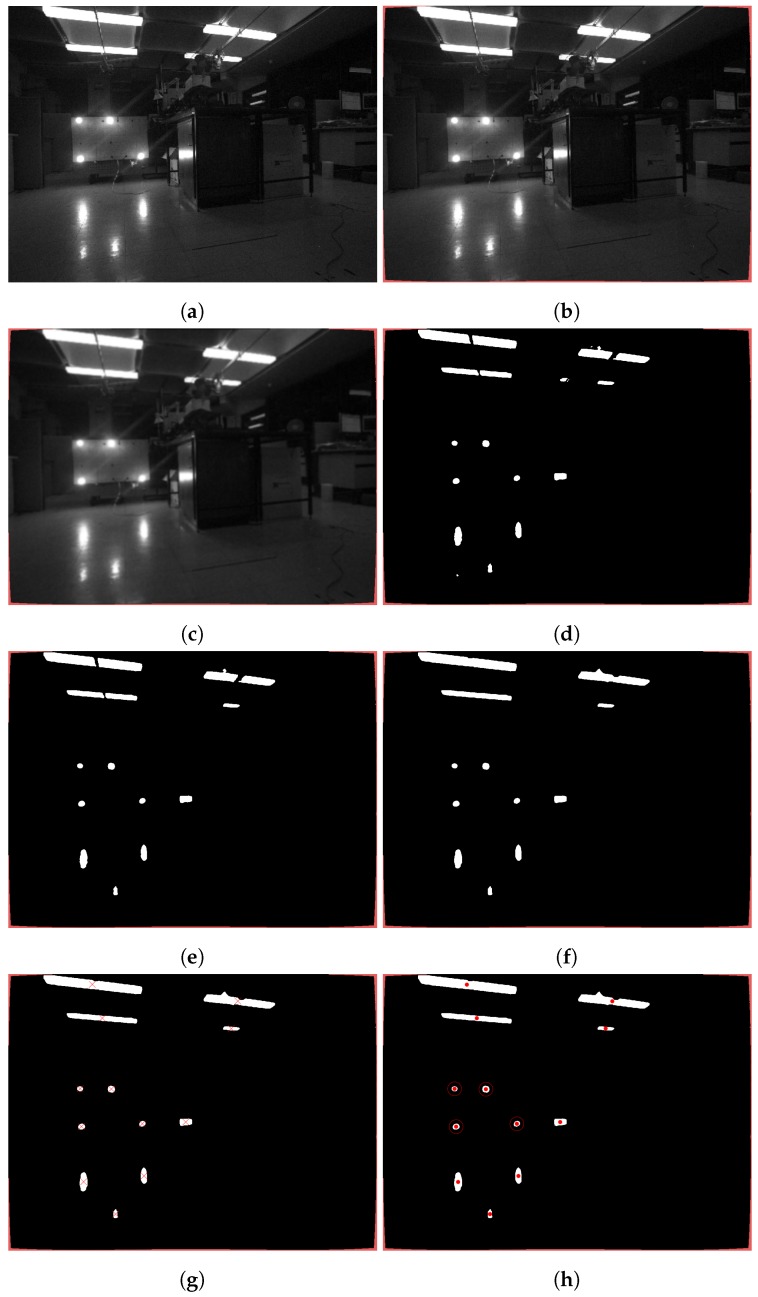
Image processing stages. Image acquisition (**a**); image undistorted (**b**); image blurred (**c**); image binarization (**d**); objects with less than P pixels removed (**e**); image morphologically closed (**f**); finding centres of remaining object and calculating object roundness (**g**); four roundest objects chosen (**h**).

Figure 5 shows the image captured during the algorithm testing on dry in the laboratory. As shown in the figure, light conditions during the algorithm testing were more complex than during the trials, as shown in Figure 2, since, at depths greater than 30 m, sunlight reflections off the metal parts are weak. The light marker with four light beacons is in the centre of the frame. The reflections of the marker light beacons are visible on the floor and the cabinet on the right, next to the marker. The marker light is slightly scattered, and multiple sources of the light are on the ceiling of the room. The image is first undistorted and blurred to partially soften reflections and scattered light (b,c). The image is then binarized as shown in Figure 5d, and objects that have fewer than P pixels are removed (e). The size of P is determined experimentally. In the next step, the image is morphologically closed (f), which fills the gaps between two objects separated by less than N pixels. However, by moving the marker further away from the camera, the distance between the light beacons mapped in the camera frame is reduced. Therefore, the N value must be less than the distance between the two closest light beacons in the camera frame at any time. In the last step, the centres of the remaining objects’ mass are calculated (g), and the roundness of the objects is checked. The four roundest objects are then detected and chosen as candidates for pose estimation (h).

### 4.2. Pose Estimation

Given the coordinates of light beacons $B^{M}$ in marker frame, their corresponding image coordinates $I_{B}$, and intrinsic camera parameters K acquired during calibration process, the extrinsic camera parameters are calculated as:

(1) $$\left[R_{\mathrm{Cam}}^{M},t_{\mathrm{Cam}}^{M}\right]=E\left(B^{M},I_{B},K\right),$$

where $B^{M}$ is an M×3 matrix with at least M=4 coplanar points, $I_{B}$ is a corresponding M×3 matrix, $R_{\mathrm{Cam}}^{M}$ is a 3D rotation matrix, and $t_{\mathrm{Cam}}^{M}$ is a 3D translation vector.

**Figure 6:**
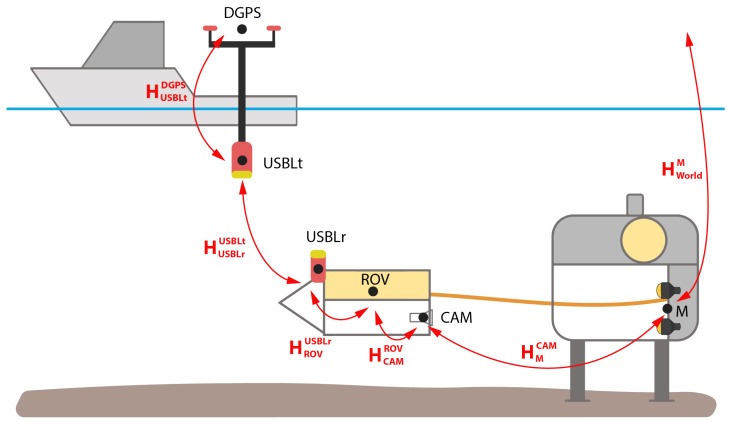
Homogeneous transformations between ship, ROV, TMS and world coordinate frames.

Figure 6 shows the relevant homogeneous transformations between the ROV and the world frame. To validate the performance of the vision pose estimation, the ROV position was measured simultaneously with the USBL system. The USBL position measurements are considered the ground truth position data.

The output of the visual pose estimation is the homogeneous transformation between the light marker and camera $H_{\mathrm{Cam}}^{M}$ consisting of relative position vector $t_{\mathrm{Cam}}^{M}$ and orientation matrix $R_{\mathrm{Cam}}^{M}$. The position of the ROV in the world frame is calculated as:

(2) $$H_{\mathrm{world}}^{\mathrm{ROV}}=H_{\mathrm{Cam}}^{\mathrm{ROV}}H_{M}^{\mathrm{Cam}}H_{\mathrm{world}}^{M},$$

where the transformation matrix $H_{\mathrm{Cam}}^{\mathrm{ROV}}$ describes the relative position and the orientation between the ROV and the camera frame. The transformation matrix between the camera and the light marker $H_{M}^{\mathrm{Cam}}$ is acquired using a visual pose estimation. Since TMS with light beacons presents permanently deployed subsea structure, the position and orientation of the marker in the world frame $H_{\mathrm{world}}^{M}$ is measured prior to experiment; therefore, it is known, and it is assumed that it was not changed over time.

**Figure 7:**
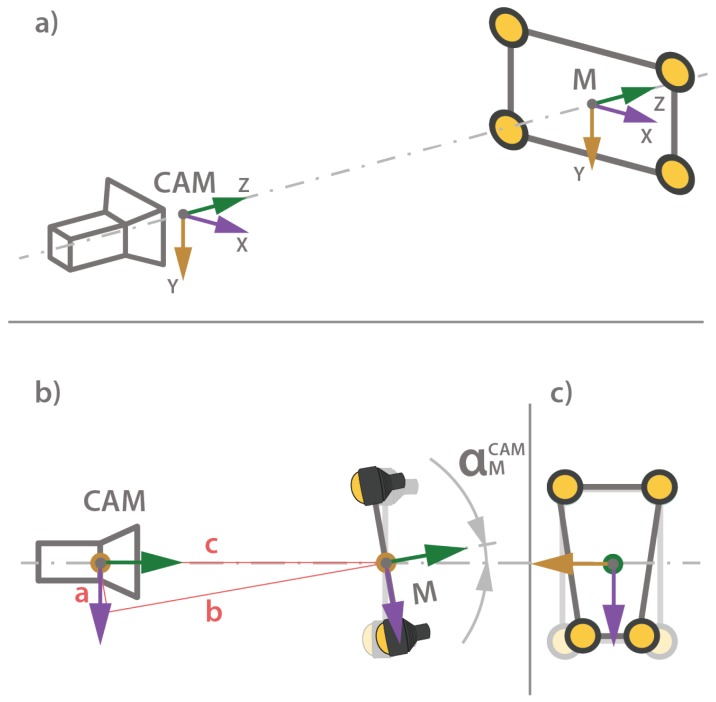
The camera and light marker coordinate systems (**a**), relative heading $\alpha_{M}^{\mathrm{Cam}}$ between the image acquisition camera and light marker (**b**), maps as a perspective distortion of light beacons in camera projection plane (**c**).

This method showed excellent pose/position estimation when the distance between the marker and the camera is less than 3.5 m, and sufficient accuracy for navigation when the position is estimated from a more significant distance out as far as 10 m distance to marker [25]. As shown in Figure 7, during the visual pose estimation process, a relative heading between the marker and the camera maps as a perspective distortion of the marker in the camera projection plane. If the distortion is not detected correctly, the rotation matrix $R_{\mathrm{Cam}}^{M}$ contains angle measurement errors. From a longer distance, those relatively small angle measurement errors can produce a significant error in the pose estimation. Since the orientation of the marker in the world frame is known, and the ROV attitude is measured with high precision IMU, to compensate for the angle measurement error, the relative orientation between the camera and marker ${R_{r}}_{\mathrm{Cam}}^{M}$ can be derived.

The corrected position of the marker in the camera coordinate frame is calculated as:

(3) $$p_{\mathrm{Cam}}^{M}=t_{M}^{\mathrm{Cam}}{R_{r}}_{\mathrm{Cam}}^{M}.$$

As discussed in the previous section, the position estimation accuracy in pure inertial mode depends on the accuracy of the accelerometers and the FOGs, and accuracy of the initial position measurement. While the accuracy of the IMU data depends on the quality of the sensor, thus cannot be improved, and more accurate position estimation enables better accuracy of the position measurement.

## 5. Results

The methods presented in Section 3 and Section 4 have been used for position estimation and position error propagation prediction in a real-world environment test. Prior to the test, the TMS system with light-based marker was deployed to the seabed. Since the INS operates in the world frame, it was necessary to determine marker position in the world frame.

After the TMS deployment, the ROV was docked and latched into the TMS. Based on the known ROV position within the TMS when docked and latched, the ROV navigation system was used to measure the TMS position and the orientation in the world frame. The position and the orientation of the light marker in the TMS frame were measured prior to the deployment. Thus, the position of the light marker in the world frame was derived.

### 5.1. Visual Pose Estimation—Static Test

**Figure 8:**
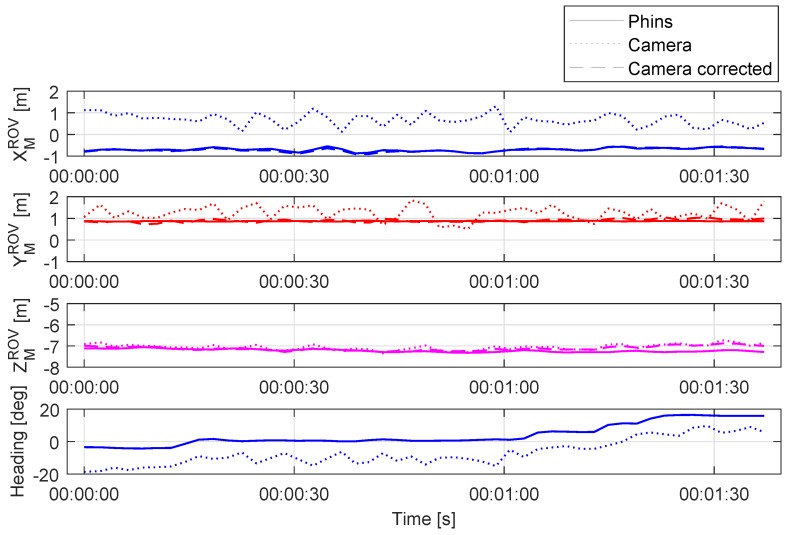
Position estimation of the camera in marker coordinate frame during the static test.

Figure 8 shows the ROV position estimation in the $X_{M}^{\mathrm{ROV}}$, $Y_{M}^{\mathrm{ROV}}$, $Z_{M}^{\mathrm{ROV}}$ axis, and relative heading $\alpha_{M}^{\mathrm{ROV}}$ in the marker reference frame. The coordinate frames were shown previously in Figure 7. During the test, the ROV was approximately 7.1 m from the light marker. The ROV was holding position while heading was changed in increments of 5 degrees, for a total change of 20 degrees, as shown in Figure 8. The depth of the vehicle was constant. The continuous line presents PHINS measurements of the position and angles. The INS operated in the highest precision mode with all external sensors used (USBL, DVL, depth sensor). The PHINS measurements are considered ground truth with position standard deviation during the test between 0.2 m and 0.3 m. The heading standard deviation was 0.04 deg, while roll and pitch standard deviation was 0.001 deg. The dotted line shows visual pose estimation measurements. As shown, the visually estimated pose data are noisy and contains significant errors within $R_{\mathrm{Cam}}^{M}$. After the rotation matrix is replaced with ${R_{r}}_{\mathrm{Cam}}^{M}$, the new position is calculated. The dashed line presents the updated ROV position based on fusion of PHINS relative angle measurements and camera estimated position.

**Figure 9:**
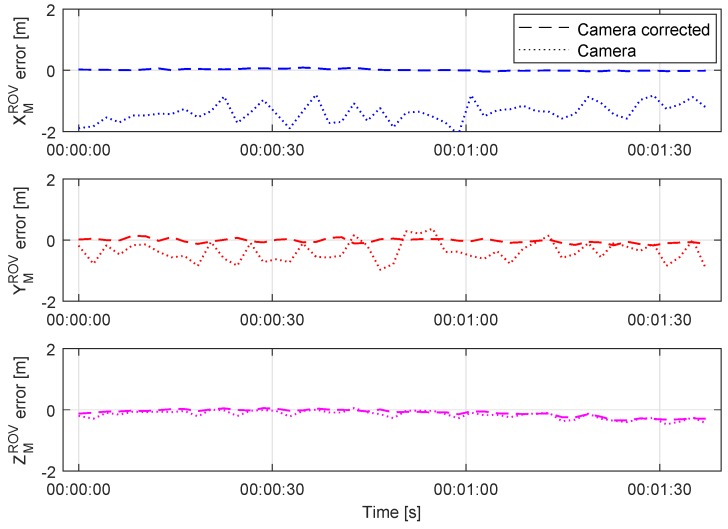
Relative position error of visually estimated pose before and after correction during the static test.

**Figure 10:**
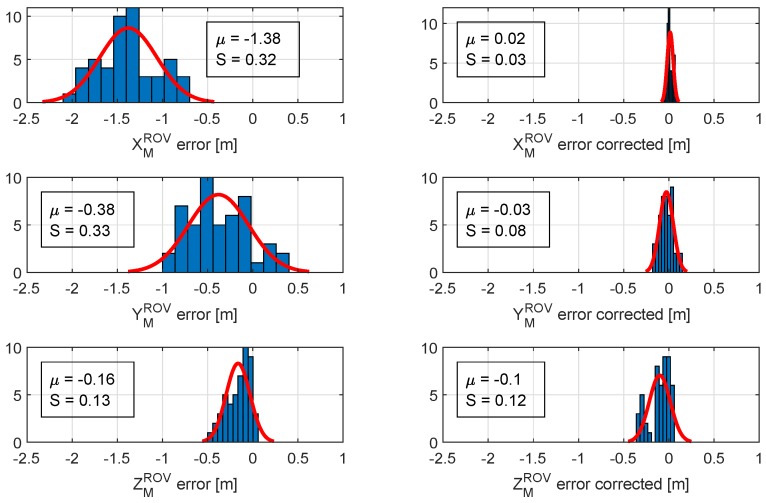
Position error distribution in marker frame before (**left column**) and after correction (**right column**).

As shown in Figure 9, the position error is significantly reduced. As expected, the IMU angle measurements outperform the visually estimated relative orientation. The measurements contain less noise, and the updated position is more accurate. Figure 10 shows position error distribution and mean values in the XYZ axes with a normal distribution curve fitted. The graphs in the left column present the distribution of the visually estimated relative pose error before the position correction. The right column shows the error distribution after the IMU angle measurements were used for a position correction. As shown, the mean error value and standard deviation are significantly reduced. The biggest improvement is achieved in $X_{M}^{\mathrm{ROV}}$ and $Y_{M}^{\mathrm{ROV}}$, since those estimations mostly depend on the perspective distortion of the light marker in the camera frame, thus relative angle measurements.

The error in $Z_{M}^{\mathrm{ROV}}$ axis is reduced as well; however, the improvement is less noticeable due to the small estimation error prior to position correction. Since the distance from the marker on the *z*-axis is estimated by calculating the relative distance between the light beacon image coordinates, the position estimation on the *z*-axis is less prone to error measurements. In addition, the error on the *z*-axis is less affected by errors in relative heading $\alpha_{M}^{\mathrm{Cam}}$, as shown in Figure 7b), since for relative heading errors $\alpha_{M}^{\mathrm{Cam}}$ = ±10 deg, the distance c≈b.

The experiment showed that visual pose estimated data are comparable with data acquired by PHINS operating in highest precision mode. The vision system performed well in good visibility up to 10 m from the target using light beacon-based position marker and with the standard deviation less than 0.5 m.

### 5.2. Visual Pose Estimation—Dynamic Test

**Figure 11:**
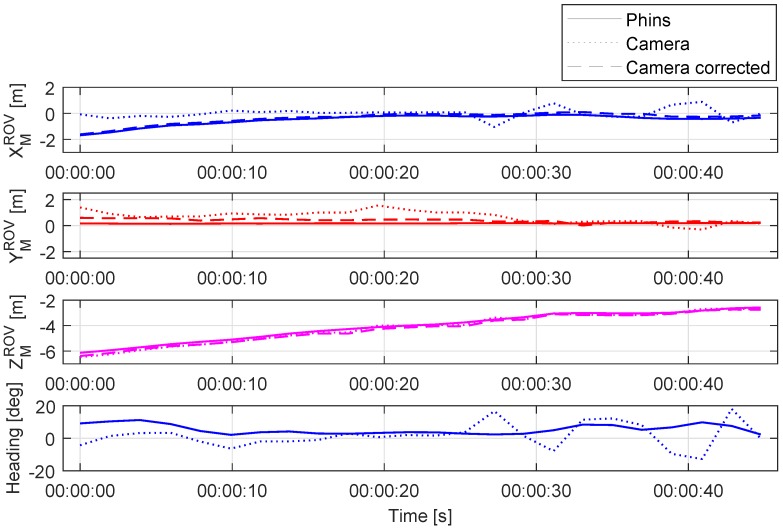
Position estimation of the camera in marker coordinate frame during the dynamic test.

The ROV position and relative heading in the M frame during a dynamic test are shown in Figure 11. The test begins with the ROV placed approximately 6 m from the light marker in $Z_{M}^{\mathrm{ROV}}$ axis, and approximately 2 m in the $X_{M}^{\mathrm{ROV}}$ axis. After the initial ROV position is measured, the vehicle is sent to the position approximately two meters from the marker $Z_{M}^{\mathrm{ROV}}$=2 m, and aligned with the marker frame in $X_{M}^{\mathrm{ROV}}$ axis. The ROV depth has been constant throughout the experiment. As the vehicle approaches the marker, at the time around 25 s and distance $Z_{M}^{\mathrm{ROV}}$≈3.5 m from the marker, the camera estimated position and heading (dotted line) starts to overlap with the PHINS position (continuous line). While during the static test shown in Figure 8 it may seem that the camera estimated relative heading has a constant offset from the PHINS heading, Figure 11 shows that the offset changes with the distance from the marker, and, as the ROV gets closer to the marker, the camera-based pose estimation becomes more accurate.

**Figure 12:**
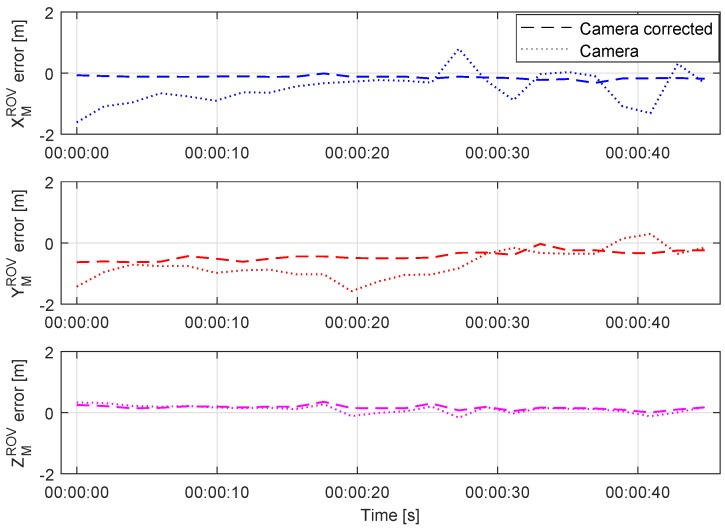
Relative position error of visually estimated pose before and after correction during the dynamic test.

**Figure 13:**
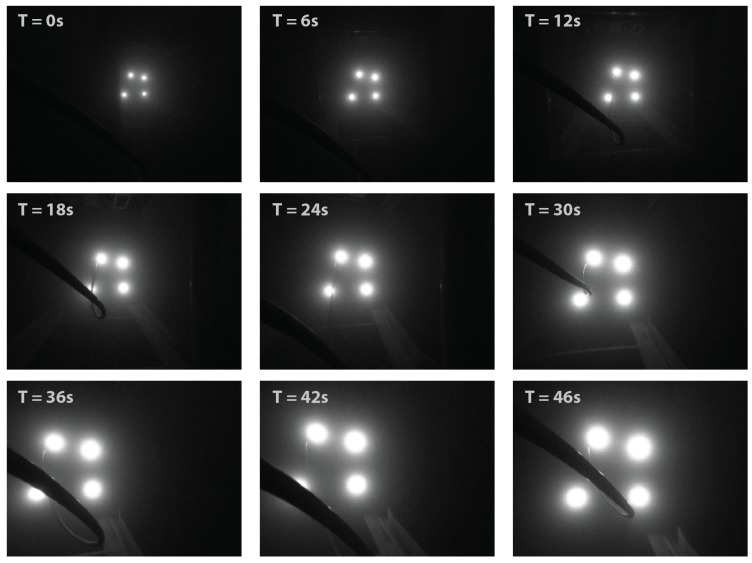
The dynamic test. The ROV position is estimated and corrected while ROV is approaching the light marker.

However, to achieve more accurate camera pose estimation throughout the whole distance range, the IMU angle measurements have to be used. As shown in Figure 12, the position error has been reduced and improved in all axes, with the significant improvement achieved in $X_{M}^{\mathrm{ROV}}$ and $Y_{M}^{\mathrm{ROV}}$ as expected. Figure 13 shows a series of images during the dynamic test as seen from the image acquisition camera. The measurement noise caused by partial light marker occlusion, most visible in heading measurement in Figure 11, has been significantly reduced after the camera pose estimation correction using the IMU angle measurements. The problems associated with the light marker coverage have been previously addressed and discussed in more detail in [25]. However, the partial light marker occlusion due to the ROV tether is shown in Figure 13, at time T = 42 s.

### 5.3. Visual Pose Estimation for INS Position Update

The experiment simulates a hypothetical scenario of the autonomous vehicle completing a dock at docking station within resident field. The initial conditions are as follows:

- The vehicle has no USBL onboard and is travelling at cruising speed with navigation system based solely on INS.
- During the trial, the vehicle DVL system is disabled in order to effect a faster integration drift in INS system over time than just 10 minutes operation with DVL.
- The vehicle operates in a structured environment.
- Approximately halfway to the docking station, there is a known landmark which can be recognized with the onboard vision system, and the position and orientation of landmark is known in world frame.

**Figure 14:**
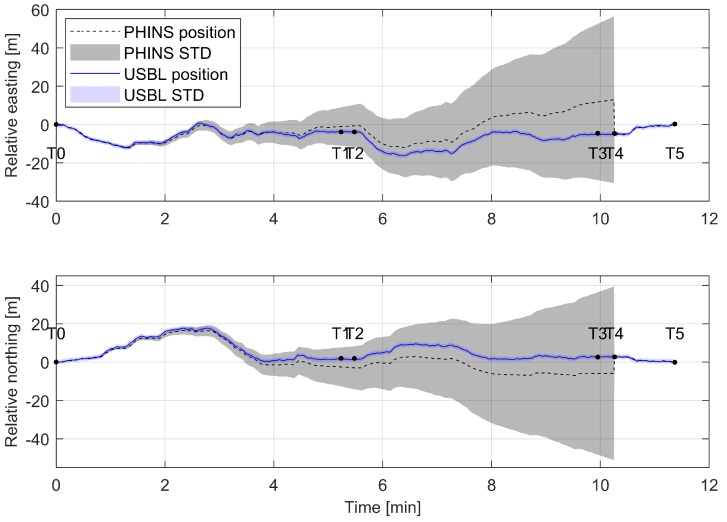
Relative ROV position throughout the experiment. Blue continuous line presents the ROV ground truth USBL position. The PHINS estimated position, while operating in pure inertial mode, is shown with black dashed line. At time T4, PHINS was switched to operate in INS + USBL mode, and the ROV was docked at T5.

The experiment starts at time T0 with the ROV docked and the navigational system running in USBL + INS mode. The INS position standard deviation is low. After the position STD reached a minimum value of 0.26 m, PHINS was switched to operate in pure inertial mode, which simulates the USBL signal loss. The ROV was then piloted for approximately 10 minutes in the area around the TMS. Since the propagation of position error in pure inertial mode does not depend on travelled distance or speed, to reduce the risk of damaging the vehicle, the ROV was piloted in relatively close proximity to the deployed TMS. Figure 14 shows relative northing and easting between the ROV and the light marker during the experiment.

The black dashed line shows PHINS estimated position throughout the experiment while the grey shaded area presents the corresponding position standard deviation. During the experiment, the ROV position was simultaneously measured with the USBL system. The blue continuous line shows the ground truth, USBL measured ROV position with corresponding position standard deviation. To avoid possible noisy measurements and achieve highest precision, the supporting vessel with the USBL transponder was positioned in direct ROV line of sight at all times. Due to the exponential nature of the position error propagation, the position error propagates slowly at the beginning. As shown in the figure, at time T1 after approximately 5 min, the PHINS position standard deviation is around 10 m, reaching over 40 m after 10 min at T4. At time T4 the PHINS system was switched to operate in INS + USBL mode, thus the position error standard deviation (gray shaded area) dropped to an initial value, and the ROV position was corrected. The ROV was then docked at T5, and the experiment finished.

**Figure 15:**
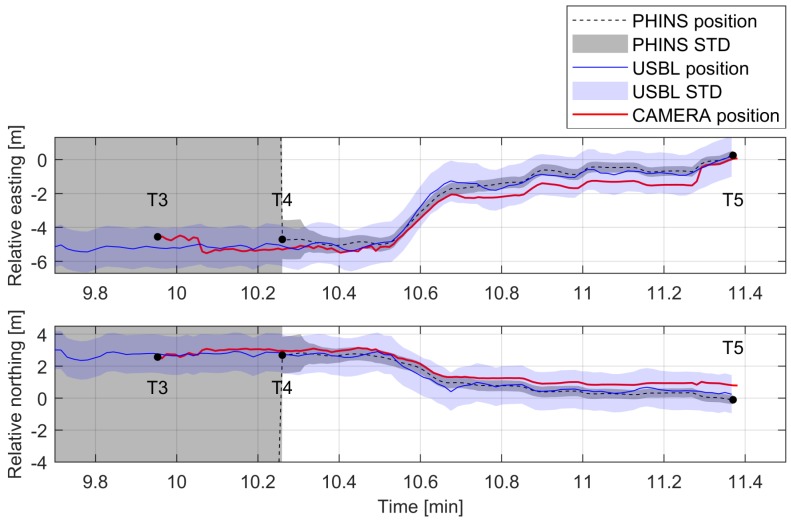
The comparison between PHINS data, USBL data, and visually estimated pose acquired during the trials.

Throughout the experiment, the position of the ROV was visually estimated two times. Between time T1–T2, which presents ROV passing by a known landmark, and between T3–T5, upon reaching the docking station. The magnified section of Figure 14 between time T3–T5 is shown in Figure 15, and it shows the comparison between, PHINS data, USBL data, and visually estimated pose data. The figure shows that the visually estimated pose (red continuous line) is comparable with the USBL data (blue continuous line). Therefore, in case of USBL signal loss, while PHINS operates in pure inertial mode, the visual pose estimation data could be used as the intermittent INS position update to reduce the position error and to allow for transition of the subsea vehicle towards another known landmark or area covered with the USBL signal.

The influence of intermittent, vision based position update on position error estimation was further investigated. Since PHINS 6000 does not provide a designated input for a visually acquired position update, the visually estimated ROV position between T1–T2 and T3–T5 could not be fed into the system Kalman filter. However, the known initial position, and the error propagation function modelled in Section 3, allows for estimation of the family of possible position trajectories and corresponding position error.

**Figure 16:**
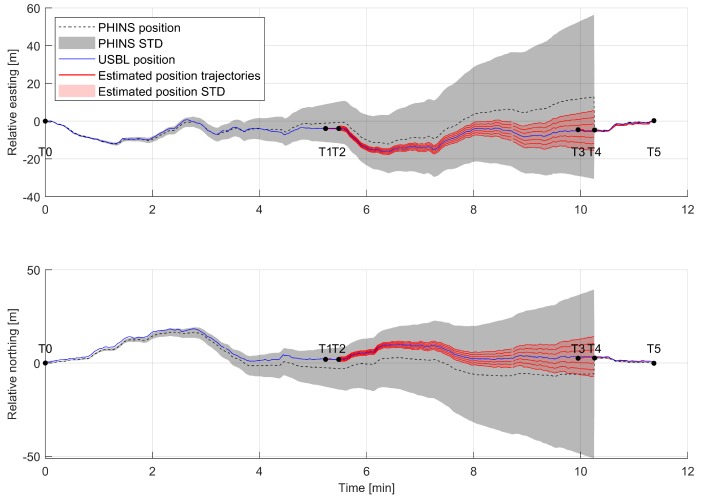
Family of position estimation trajectories (red continuous lines) from time T2 to T4 in case of ROV position update between time T1 and T2.

Figure 16 shows a family of possible position estimation trajectories between time T2 and T4 (continuous red lines) in case the ROV position was updated between T1–T2 and the ROV continued to operate in pure inertial mode until T4. A specific position trajectory could not be simulated since PHINS 6000 exact mathematical model of the EKF, sensor error models, and the raw IMU sensor data are not available. A red shaded area presents the estimated position STD between T2 and T4, in case of position update at T2, for the trajectory which aligns with the ground truth data.

## 6. Discussion and Conclusions

This paper shows experimental results of a vision-based localization system for the resident ROVs/AUVs which can be utilised to eliminate drift error from on-vehicle inertial navigation system. The proposed system is developed around the standard equipment found throughout the ROV sector. The vision estimated pose based on active light-based marker recognition showed good overall performance and is comparable with the measured USBL ground truth position. While INS operates without USBL/LBL, the visually estimated pose could be used as a position fix, thus reducing the position error caused by position drift.

The system has been tested in the North Atlantic Ocean during trials in January 2019. The propagation of position error in pure inertial mode was measured and modelled as a function of time and initially estimated position error. This function is used to simulate a family of possible position trajectories in the case that vision-based position fix is acquired during the test. The visually-based pose estimation method, with IMU relative angle correction, was used to determine the relative pose between the ROV and deployed subsea asset. The results showed that the proposed system performed well and can improve the ROV/AUV localization underwater. The performance of the vision system depends on the water turbidity, in clear water providing up to a 10 m range and solution results in a low cost and stable platform for localization while not being prone to noise pollution as acoustic navigation systems. With the high water turbidity, the system operation range becomes limited and the UUV navigation purely relies on the acoustic based pose estimation technology.

High precision INS system with DVL aiding provides a strong platform; however, with the complexities involved in AUV based IMR activities, this is not accurate enough for the transition of resident systems between assets or for close quarter operations on subsea installations. The position drift of such configuration is 0.1% of the travelled distance. However, in close proximity to the marker, visual pose estimation is shown to be a reliable system for an absolute position fix. Since resident ROVs/AUVs operate in the structured environment, this low-cost solution can provide an alternative and more cost effective solution to high-maintenance acoustic-based positioning systems.

Future work on this project is investigating the input of real-time 3D dense reconstruction and tracking system, known as SteroFusion [27], as sensor aiding to INS to improve subsea navigation.

## Figures and Tables

**Table 1:** The DVL, USBL, and GPS system technical specification.

**Nortek 500 DVL**	
Bottom velocity	
Single ping std @ 3m/s	5 mm/s
Long term accuracy	±0.2%/±1 mm/s
Minimum altitude	0.3 m
Maximum altitude	200 m
Velocity resolution	0.01 mm/s
Current profiling	
Minimum accuracy	1% of measured value / ±5 mm/s
Velocity resolution	1 mm/s
**Teledyne Ranger 2 USBL**	
Operating range	>6000 m
System accuracy	0.2% of Slant range
Position update rate	1 s
**Okeanus DGPS**	
Position accuracy GPS	<15 m
Position accuracy DGPS (WAAS)	<3 m
PPS Time	±1 us

**Table 2:** The PHINS 6000 INS system technical specification.

Position accuracy with USBL/LBL	Three times better than USBL/LBL accuracy
Position accuracy with DVL	0.1% of travelled distance
Position accuracy with no aiding for 1 min/ 2 min	0.8 m/ 3.2 m
Heading accuracy with GPS	0.01 deg secant latitude
Heading accuracy with DVL/USBL/LBL	0.02 deg secant latitude
Roll and Pitch accuracy	0.01 deg
Heave accuracy	5 cm or 5% (Whichever is greater)

## Abbreviations

The following abbreviations are used in this manuscript:

AUV	Autonomous unmanned vehicle
CMOS	Complementary metal-oxide-semiconductor
DVL	Doppler velocity log
EKF	Extended Kalman filter
FOG	Fibre optic gyroscope
GPS	Global positioning system
IMR	Inspection, maintenance and repair
IMU	Inertial measurement unit
INS	Inertial navigation system
LARS	Launch and recovery system
LBL	Long baseline
LCOE	Levelized cost of energy
LED	Light emitted diode
MRE	Marine renowable energy
OPEX	Operational expenditure
ROV	Remotely operated vehicle
RV	Research vessel
SLAM	Simultaneous localization and mapping
STD	Standard deviation
TMS	Tether management system
USBL	Ultra-short baseline
